# Comparing the cohort design and the nested case–control design in the presence of both time-invariant and time-dependent treatment and competing risks: bias and precision

**DOI:** 10.1002/pds.3299

**Published:** 2012-06-01

**Authors:** Peter C Austin, Geoffrey M Anderson, Candemir Cigsar, Andrea Gruneir

**Affiliations:** 1 Institute for Clinical Evaluative SciencesToronto, Ontario, Canada; 2 Institute of Health Management, Policy and Evaluation, University of TorontoToronto, Ontario, Canada; 3 Dalla Lana School of Public Health, University of TorontoToronto, Ontario, Canada; 4 Women's College Research InstituteToronto, Ontario, Canada; 5 Department of Biostatistics, Princess Margaret HospitalToronto, Ontario, Canada

**Keywords:** observational study, cohort design, nested case–control design, case–control design, Monte Carlo simulations, bias, precision, pharmacoepidemiology

## Abstract

**Purpose:**

Observational studies using electronic administrative healthcare databases are often used to estimate the effects of treatments and exposures. Traditionally, a cohort design has been used to estimate these effects, but increasingly, studies are using a nested case–control (NCC) design. The relative statistical efficiency of these two designs has not been examined in detail.

**Methods:**

We used Monte Carlo simulations to compare these two designs in terms of the bias and precision of effect estimates. We examined three different settings: (A) treatment occurred at baseline, and there was a single outcome of interest; (B) treatment was time varying, and there was a single outcome; and C treatment occurred at baseline, and there was a secondary event that competed with the primary event of interest. Comparisons were made of percentage bias, length of 95% confidence interval, and mean squared error (MSE) as a combined measure of bias and precision.

**Results:**

In Setting A, bias was similar between designs, but the cohort design was more precise and had a lower MSE in all scenarios. In Settings B and C, the cohort design was more precise and had a lower MSE in all scenarios. In both Settings B and C, the NCC design tended to result in estimates with greater bias compared with the cohort design.

**Conclusions:**

We conclude that in a range of settings and scenarios, the cohort design is superior in terms of precision and MSE. Copyright © 2012 John Wiley & Sons, Ltd.

## INTRODUCTION

There is an increasing interest in using large administrative healthcare databases for comparative effectiveness, epidemiological, and pharmacoepidemiological research. Advantages to the use of administrative healthcare databases include comprehensive coverage of entire populations, relatively low cost for the acquisition of data on outcomes and covariates, and the ability to examine the effects of treatments and interventions as they are applied outside of the tightly controlled confines of randomized controlled trials.

The traditional approach to the analysis of these large observational datasets is the retrospective cohort design. The treatment status of each subject is determined at the time of cohort entry or at some observable time subsequent to cohort entry. Subjects are followed over time for the occurrence of the outcome of interest. The incidence of the outcome is then compared between those who were treated and those who were untreated using the incidence rate ratio.

In observational studies, treatment assignment is not at random but is often influenced by subject characteristics. There are often systematic differences in baseline characteristics between treated and untreated subjects. Therefore, statistical methods must be used to reduce the bias in the estimate of association. For a cohort design with time-to-event data, this is often accomplished using the Cox proportional hazards model.^1^ The estimated hazard ratio represents the adjusted incidence rate ratio.

There has been a recent increase in the use of the nested case–control (NCC) design in pharmacoepidemiological studies.^2^–^12^ The NCC is a variant of the classic case–control design where cases and controls are sampled from a well-defined cohort.^13^–^17^ The measure of association derived from any case–control study, the odds ratio, is mathematically equivalent to the incidence rate ratio derived from a cohort study given that the ratio of treated to untreated in the control series is equivalent to the ratio of the treated to untreated person-time in the source population.^18^ Traditional arguments in favor of the case–control design focused primarily on its improved efficiency relative to the cohort design. In this context, efficiency was defined as the potential to reduce the costs and/or burden of data collection. This efficiency arises from the fact that, whereas in a cohort study, data on covariates must be collected from all subjects, in a case–control design, data on covariates are required from all cases, but from only a sample of those who do not experience the outcome (i.e., the controls). This is not relevant in studies using administrative or other secondary data where the marginal cost of data collection for covariates is close to zero. More recently, some authors have suggested that another form of efficiency relates to computational efficiency, in particular, where there may be some time-varying element to the treatment.^19^ Given the ongoing increases in computational speed and processing power, this aspect of efficiency may be less relevant for many analyses.

Because identifying a well-defined cohort is the first step when using either a cohort or NCC design, it is possible to use either design to estimate treatment effects in the same set of subjects. Although both designs, given specific conditions, can result in unbiased treatment effects when the research question relates to a relatively simple treatment–outcome relationship, it is less clear how these designs compare when more complex treatment–outcome relationships are of interest. Understanding the implications of one design over the other is required for investigators to make informed decisions. One way to judge the comparative quality of the estimates of treatment effect produced by the two designs is the bias and precision of these estimates.

The objective of the current study was to compare estimates of treatment effect made from a cohort design with those from an NCC design in terms of bias and precision. We used a series of Monte Carlo simulations to examine these issues in three different settings that describe important treatment–outcome relationships in pharmacoepidemiology: Setting A, the least complex setting, in which there is a single event of interest and subjects are treated/exposed at baseline and treatment status remains fixed over the duration of follow-up time. Setting B introduces a variation in the definition of treatment, with treatment status being allowed to vary over time. However, there is still only one event of interest. Setting C introduces an issue related to the outcome by allowing there to be secondary outcomes or events, which compete with the primary event of interest. In each of the three settings, we examined several different scenarios defined by the magnitude of the true treatment effect, the proportion of subjects who were treated, and the proportion of subjects who experienced the event or outcome.

## MONTE CARLO SIMULATIONS—DESIGN

The operational definition of exposure varies widely across studies that use the NCC design. We examined three simple settings that form a foundation for more complex definitions of exposure. First, we considered a setting in which exposure is applied at the time of cohort entry and remains fixed over the duration of follow-up. Examples of this include studies comparing the effect of different chemotherapy regimes on patients diagnosed with specific cancers. A second example is a study to examine serum levels of superoxide dismutase activity and the risk of cancer mortality, in which the base cohort was the Japan Collaborative Cohort Study.^20^ Cases were subjects who died of cancer. Exposure was defined using blood serum donated close to the time of cohort entry. The second setting we considered involved a point exposure that was applied at some point during the duration of follow-up. Examples of this include studies in which vaccines may have been administered at some point after cohort entry. Another is an Australian study to examine the effect of anti-inflammatory drugs on the incidence of myocardial infarction and all-cause mortality in the Australian veteran community.^21^ Cases were subjects who experienced myocardial infarction, heart failure, or death of any cause. In the first reported analysis, exposure was defined as any receipt of a nonsteroidal anti-inflammatory medication during the follow-up period. The third scenario that we considered is an adaption of the first scenario, but with the outcome being a nonmortality outcome that is subject to competing risks (e.g., hip fracture, hospitalizations, or occurrence of a specific disease).

### Data-generating process

We used a series of Monte Carlo simulations to examine bias and precision of estimates from cohort designs with those from NCC designs. We examined the following three settings: Setting A: a binary treatment was assigned and fixed at baseline; Setting B: a binary treatment was assigned at some time during the duration of the follow-up; and Setting C: similar to Setting A, but there were competing events. The basic setup of the simulations was similar across the three settings. These settings are important because they form the basis for more complex methods of defining exposure.

For a given iteration of the Monte Carlo simulation, we simulated baseline covariates, treatment status, and an outcome for each of 5000 subjects. For each subject, we simulated six baseline covariates (*X*_1_–*X*_6_), the first three from independent Bernoulli distributions with parameter 0.5 and the last three from independent standard normal distributions.

In Settings A, B, and C, we determined a treatment status at baseline using a logistic regression model:


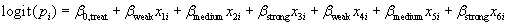
(1)

The values of *β*_weak_, *β*_medium_, and *β*_strong_ were set to log(1.10), log(1.50), and log(2), respectively, to denote weak, medium, and strong treatment selection effects. The value of the intercept, *β*_0,treat_, was selected so that the marginal probability of receipt of treatment would be fixed at the desired level (this was one of the factors of the Monte Carlo simulations). We then simulated a treatment status from a Bernoulli distribution with subject-specific parameter *p_i_*. For Settings A and C, treatment status was assigned at baseline and then fixed over the duration of follow-up. In Setting B, for those subjects who were assigned to receive treatment, time to receipt of treatment was randomly generated from a Weibull distribution with shape and scale parameters of 0.25 and 433.2097, respectively; thus, the median time to treatment would be 100 days. We thus generated a treatment status for each subject and in Setting B, a time at which treatment was to be received.

We then simulated a time-to-event outcome for each subject using a Cox–Weibull model. For Setting A, in which treatment selection was fixed at baseline, a previously described data-generating process^22^,^23^ was used to simulate time-to-event outcomes from the following Cox model:


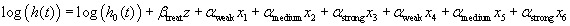
(2)

where *h*_0_(*t*) denotes the baseline hazard function, and *z* is an indicator variable denoting treatment status. The values of *α*_weak_, *α*_medium_, and *α*_strong_ were set at log(1.25), log(2), and log(3), respectively, to denote weak, medium, and strong effects on the hazard of the outcome. The coefficient *β*_treat_, which denotes the log–hazard ratio for the effect of treatment on the hazard of the outcome, is one of the factors that will be varied in the Monte Carlo simulations. In each setting, we assumed a Weibull distribution for time-to-event outcomes, with shape and scale parameters of 0.45 and 0.01, respectively. If the entire population were untreated, this would result in a marginal distribution of event times with a median of approximately 1000 days and a 25th percentile of approximately 53 days.

In Setting B, in which treatment status was time dependent, a time-to-event outcome was simulated for each subject using a data-generating process described elsewhere.^24^ The same shape and scale parameters were used as stated earlier so that the marginal distribution of event times under lack of treatment was the same as in Setting A. If subjects experienced the event of interest prior to the time of receipt of treatment, the subject was defined to have been untreated for the entire duration of follow-up.

In Setting C, in which there were competing risks, a time-to-event outcome was simulated for each subject using a data-generating process described by Beyersmann *et al*.^25^ In this setting, we assumed that there were two competing events (the primary event of interest and a competing event). Furthermore, we assumed that each of these two events had the same hazard function (and that the hazard function for each event was the same as that from Setting A). Thus, the overall hazard function of either event occurring was twice the cause-specific hazard function of the primary event of interest. For each subject, using the approach described by Bender *et al*., we simulated a time-to-event outcome by inverting the cumulative overall hazard function. Then, because the two event types had the same hazard function, using the approach described by Beyersmann *et al*., we randomly selected which of the two types was the event type that occurred, with each event type having a probability of 0.5. We then followed a similar approach to the one given in Setting A.

### Factors of the Monte Carlo simulations

In each of the three settings, we used a full factorial design in which we allowed the following factors to vary: the true hazard ratio for the effect of treatment on the hazard of the outcome; the prevalence of treatment (the percentage of subjects who were assigned to treatment); and the proportion of subjects for whom the event was observed to occur, with the remaining subjects being subject to censoring. When inducing censoring, an event time was initially simulated for all subjects as described in the previous section. We then determined the appropriate percentile of survival or event times. All subjects with event times that exceeded this percentile of event time were then treated as censored observations, with their observed survival time set to this percentile. In using this approach, we induce Type II censoring.^26^ However, this will not induce any bias in estimating regression coefficients (26; Section III.2).

In each of the three settings, the hazard ratio was allowed to take on the following values: 1.25 and 2. The proportion of subjects who were treated took on the following values: 0.10, 0.25, and 0.50, whereas the proportion of subjects for whom the event was observed to have occurred was 0.05, 0.10, and 0.25. Thus, for each of the three settings, there were 18 different scenarios (2 hazard ratios × 3 proportion of subjects treated × 3 proportion of subjects who were censored). In each of these 18 different scenarios, 1000 datasets were simulated, each consisting of 5000 subjects. In Setting C, although the proportion of subjects for whom any event was observed to have occurred took the following values: 0.10, 0.20, and 0.50 (because half of the observed events would be the primary event, whereas the other half would be the competing event, this implies that the primary event would be observed to occur for 5%, 10%, and 25% of subjects).

### Statistical analyses

In each simulated dataset, the following statistical analyses were conducted. First, an analysis based on a conventional cohort design was conducted. A Cox proportional hazards regression model was used to regress survival time on an indicator variable denoting treatment status and the six baseline covariates. In Setting A, a conventional Cox model with time-invariant covariates was fit to each simulated dataset. In Setting B, the Cox model accounted for the time-dependent nature of treatment status: For subjects who were assigned to receive the treatment, subjects were considered untreated until the time of receipt of treatment. In Setting C, a Cox model was used to model the cause-specific hazard of the primary event of interest, treating the occurrence of the competing event as a censoring event.^27^ In each case, the log–hazard ratio for the treatment effect and its standard error were estimated, along with the 95% confidence interval for the estimated hazard ratio.

Second, an analysis based on the NCC design was used. Cases were defined to be subjects who experienced the event of interest. For each case, one or more controls were selected by simple random sampling without replacement from the subjects in the case's risk set. A case's risk set is the set of subjects who were still at risk of the event at the time at which the case experienced the event of interest. Thus, each case was matched to a subject who, at the time that the case experienced the event of interest, had not yet experienced the event of interest. In Setting A, we used both 1:1 and 5:1 matching. In 1:1 matching, pairs of cases and controls were formed, whereas with 5:1 matching, each case was matched to up to five controls. Thus, for 1:1 matching, from a case's risk set, one subject was selected at random for matching to the given case; for 5:1 matching, five subjects were selected at random from the case's risk set for matching to the case. For each case, the index date was defined to be the time of the occurrence of the event of interest, whereas for each control, the index date was the time at which the event occurred for the matched case. For each case and the matched controls, we determined whether they had been treated/exposed prior to the index date. Conditional logistic regression was then used to determine the association between exposure and the occurrence of the event of interest while adjusting for the six baseline covariates and accounting for matched sets. From the conditional logistic regression model, we estimated the adjusted log–odds ratio for exposure, the standard error of the adjusted log–odds ratio, and the 95% confidence interval for the adjusted odds ratio.

For each setting and each scenario, let *θ*_i_ denote the log–hazard ratio or log–odds ratio estimated in the *i*th simulated dataset (*i* = 1,…,1000). Bias was defined as 
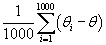
, where *θ* denotes the true log–hazard ratio used in the data-generating process. Relative bias was defined as 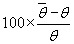, where 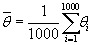. Mean squared error (MSE) was calculated as 
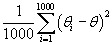
. We calculated the proportion of estimated 95% confidence intervals for the estimated hazard ratio/odds ratio that contained the true hazard ratio used in the data-generating process. Because we used 1000 simulated datasets per scenario, an empirical coverage rate that was less than 0.9365 or greater than 0.9635 would be statistically significantly different from the advertized rate of 0.95 using a standard normal-theory test. Finally, we estimated the mean width of the estimated 95% confidence intervals across the 1000 simulated datasets and compared the relative width of confidence intervals from the NCC design with those from the cohort design. Comparing the mean width of confidence intervals is equivalent to comparing the mean standard error of the estimated treatment effect from the NCC design with the mean standard error from the cohort design. Thus, this final comparison permits a comparison of the relative statistical efficiency of the two different designs.

The simulations and statistical analyses were conducted in SAS v9.2 (SAS Institute Inc., Cary, NC) and R v2.11.1 (The R Foundation for Statistical Computing, Vienna, Austria).

## MONTE CARLO SIMULATIONS—RESULTS

### Setting A—fixed exposure

**Figure 1:**
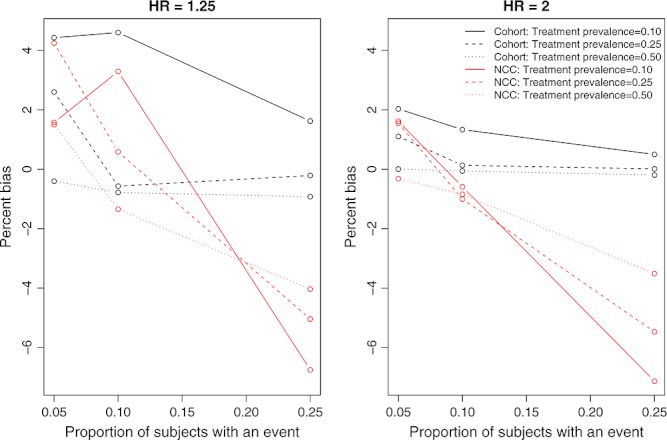
Percentage bias: fixed treatment

**Figure 2:**
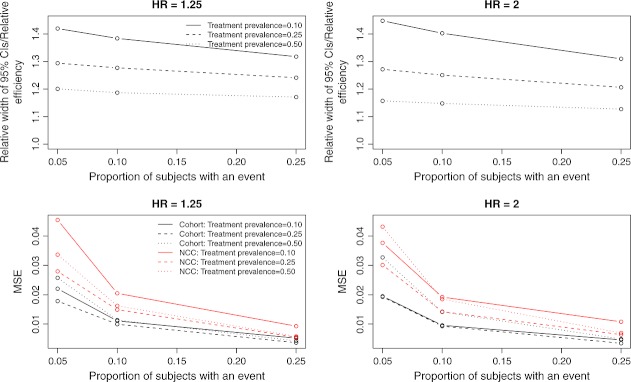
Precision/Relative efficiency and mean squared error: fixed treatment

Results for this setting are reported in Figures 1 and 2. Due to space constraints, we do not report detailed results for 1:1 matching in the NCC design; however, we summarize these results in the following two paragraphs. In Figure 1, we report relative bias. Across the 18 scenarios, the median relative bias was 0.1% for the cohort design, whereas it was 0.2% and −0.7% for the NCC analyses with 1:1 and 5:1 matching, respectively. For the cohort design, the 25th and 75th percentiles of relative bias were −0.2% and 1.6%, respectively, whereas for the NCC design with 1:1 matching, the upper and lower quartiles of relative bias were −3.4% and 2.3%. With 5:1 matching, the 25th and 75th percentiles of relative bias were −4.0% and 1.5%, respectively. In examining Figure 1, one observes that there was a trend, when using the NCC design, towards an increase in the magnitude of relative bias as the proportion of subjects for whom events were observed to have occurred increased. However, in all 18 scenarios, the relative bias tended to be small. When using the cohort design, the magnitude of relative bias tended to decrease as the proportion of subjects who were treated increased.

In the top two panels of Figure 2, we report the ratio of the mean length of the 95% confidence intervals for the NCC design with 5:1 matching to the mean length of the 95% confidence intervals for the cohort design. This is equivalent to the asymptotic relative efficiency—the ratio of the standard error of the estimate from the NCC design to the standard error of the estimate from the cohort design. The median ratio of widths of confidence intervals was 1.26 across the 18 scenarios, whereas the 25th and 75th percentiles were 1.19 and 1.32, respectively. When 1:1 matching was employed, the 25th, 50th, and 75th percentiles of this ratio were 1.60, 1.83, and 2.09, respectively. In 17 of the 18 scenarios, the empirical coverage rates from the cohort design and the NCC (with 5:1 matching) were not statistically significantly different from their advertized rates of 0.95. The inefficiency of the NCC design relative to the cohort design increased as the proportion of subjects who were treated decreased. Furthermore, the relative inefficiency of the NCC design decreased as the proportion of subjects for whom an event occurred increased.

The MSEs of the estimated treatment effects are reported in the lower two panels of Figure 2. The MSE from the cohort design was always smaller than that from the NCC design. The median MSE from the former design was 0.0105, whereas it was 0.0307 for the NCC design with 1:1 matching and 0.0173 with 5:1 matching. The MSE of the estimated treatment effect decreased as the proportion of subjects for whom an event occurred increased.

### Setting B—time-dependent treatment status

**Figure 3:**
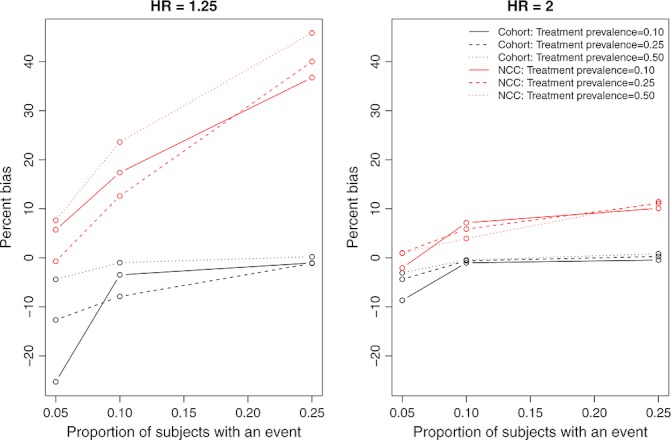
Percentage bias: time-varying treatment

The relative bias is reported in Figure 3. Across the 18 scenarios, the median relative bias was −1.1% and 8.9% for the cohort and NCC designs, respectively. For the cohort design, the 25th and 75th percentiles of relative bias were −4.4% and −0.5%, respectively, whereas for the NCC design, the upper and lower quartiles of relative bias were 3.9% and 17.4%. For the NCC design, the relative bias tended to increase as the proportion of subjects for whom an event was observed increased. When the percentage of subjects who experienced an event was low (5%) and the prevalence of treatment was either 5% or 25%, then the cohort design resulted in estimates with greater relative bias compared with the NCC design. However, in the remaining scenarios, the NCC design resulted in greater relative bias. Furthermore, relative bias tended to be greater when the true treatment hazard ratio was 1.25 compared with when it was 2. When the treatment hazard ratio was 1.25 and the event occurred for 25% of the subjects, then the relative bias could be substantial for the NCC design.

**Figure 4:**
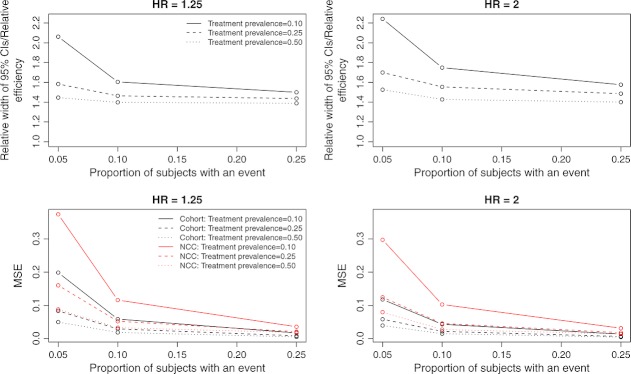
Precision/Relative efficiency and mean squared error: Time-varying treatment

In the upper two panels of Figure 4, we report the ratio of the mean length of the 95% confidence intervals for the NCC design to the mean length of the 95% confidence intervals for the cohort design. This is equivalent to the relative efficiency of the two designs: the ratio of the standard error of the NCC design to that of the cohort design. The median ratio of widths of confidence intervals was 1.51 across the 18 scenarios, whereas the 25th and 75th percentiles were 1.44 and 1.60, respectively. In 2 of the 18 scenarios, the empirical coverage rates from the cohort design were statistically significantly different from their advertized rates of 0.95. However, in 8 of the 18 scenarios, the empirical coverage rates from the NCC design were statistically significantly different from their advertized rates of 0.95. The relative inefficiency of the NCC design decreased as the proportion of subjects who experienced an event increased. The relative inefficiency also decreased as the proportion of subjects who were treated increased.

The MSEs of the estimated treatment effects are reported in the lower two panels of Figure 4. The MSE from the cohort design was always smaller than that from the NCC design. The median MSE from the former design was 0.0255, whereas it was 0.0490 for the latter. Differences between the MSEs of the estimated treatment effects from the two designs tended to decrease as the proportion of subjects who experienced an event increased and as the proportion of subjects who were treated increased.

### Setting C—competing risks

**Figure 5:**
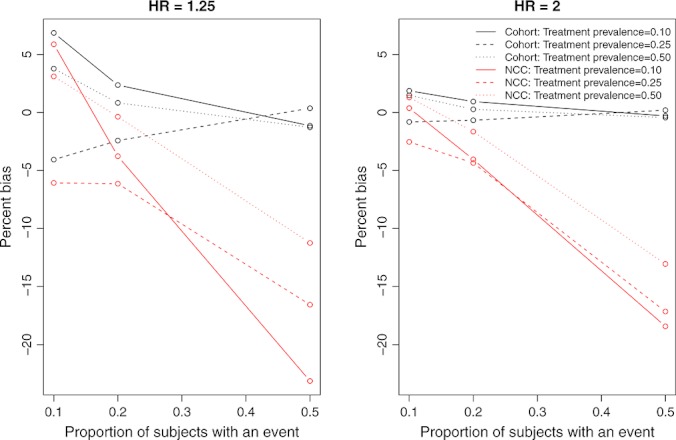
Percentage bias: competing risks

The relative bias is reported in Figure 5. Across the 18 scenarios, the median relative bias was 0.2% and −4.2% for the cohort and NCC designs, respectively. For the cohort design, the 25th and 75th percentiles of relative bias were −0.8% and 1.5%, respectively, whereas for the NCC design, the upper and lower quartiles of relative bias were −13.0% and −0.4%. When 25% of subjects experienced the event, the relative bias of the NCC design was substantial. However, when the percentage of subjects who experienced the event was low (10%), then the magnitude of the relative bias was modestly greater for the cohort design than for the NCC design.

**Figure 6:**
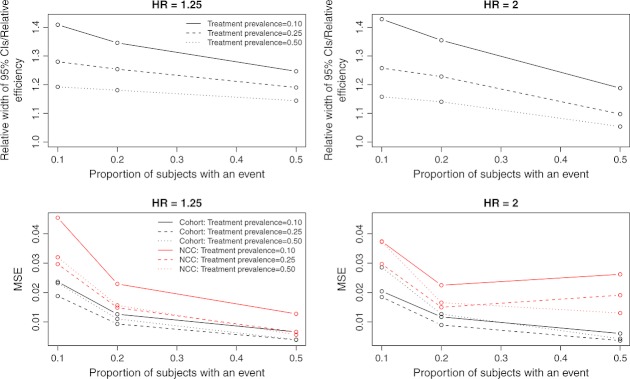
Precision/Relative efficiency and mean squared error: competing risks

In the upper two panels of Figure 6, we report the ratio of the mean length of the 95% confidence intervals for the NCC design to the mean length of the 95% confidence intervals for the cohort design. This is equivalent to the relative efficiency of the two designs: the ratio of the standard error of the NCC design to that of the cohort design. The median ratio of widths of confidence intervals was 1.21 across the 18 scenarios, whereas the 25th and 75th percentiles were 1.16 and 1.28, respectively. In none of the 18 scenarios was the empirical coverage rate of 95% confidence intervals from the cohort design statistically significantly different from the advertized rate of 0.95. However, in 5 of the 18 scenarios, the empirical coverage rates of the 95% confidence intervals from the NCC design were statistically significantly different from the advertized rate of 0.95. The relative inefficiency of the NCC design decreased as the proportion of subjects who experienced the event increased and as the proportion of subjects who were treated increased.

The MSEs of the estimated treatment effects are reported in the lower two panels of Figure 6. The MSE from the cohort design was always smaller than that from the NCC design. The median MSE from the former design was 0.0114, whereas it was 0.0208 for the latter.

## DISCUSSION

We compared the estimation of treatment effects in cohort designs using the Cox proportional hazards model with estimation in NCC designs using conditional logistic regression. When exposure was applied at time of cohort entry and in the absence of competing risks, both designs resulted in approximately unbiased estimation of log–hazard ratios. However, the cohort design resulted in estimates with greater precision and lower MSE. Thus, the penalty for using an NCC design in this context is the decreased precision or diminished statistical efficiency: The treatment effect is estimated with less precision. Thus, associated confidence intervals will be wider than if a cohort design was used. When both events or outcomes were rare and treatment or exposure occurred infrequently, then, although negligible in magnitude, the cohort design resulted in modestly more bias than did the NCC design. However, this modest increase in bias was offset by greater precision. When treatment was time dependent, then the NCC design tended to result in estimates with greater bias than those arising from a cohort design. As mentioned, the cohort design resulted in estimates with greater precision and lower MSE. Finally, in the presence of competing risks, the use of an NCC design resulted in greater bias compared with the cohort design. As with the other two settings, the cohort design resulted in estimates with greater precision and lower MSE. Thus, the primary penalty for using an NCC design rather than a cohort design is the decreased statistical efficiency, with the attendant increase in the width of estimated confidence intervals. However, in some settings, there was also an increase in the magnitude of the relative bias of the estimated treatment effect.

Our findings on bias in the setting with an exposure fixed at baseline and with no competing risks should be of no surprise. The conditional likelihood of the conditional logistic regression model is of the same form as the partial likelihood used in the Cox proportional hazards model.^28^ For this reason, one would expect the estimated log–hazard ratio and the log–odds ratio to coincide. We found that the use of a cohort design resulted in estimates of greater precision than those arising from the use of an NCC design. This is also expected, given that in the cohort design, all of the available data are used for estimation, whereas in the case–control design, only the data on cases and a sample of controls are used. When we extended our examination to settings in which time-dependent covariates and competing risks occur, we found that bias was introduced with the NCC design and that this design also resulted in estimates with less precision than that of the cohort design.

The primary rationale for conducting an NCC study even when a cohort has been assembled is to obtain additional data that would be prohibitive to collect on the full cohort (18, p. 94). Thus, as noted by Rothman and Greenland, an NCC design is more efficient than a cohort study. However, it is important to note that they are using the term *efficiency* in an economic or expenditure of effort sense and not in a statistical perspective. They suggested that an NCC study may be substantially cheaper to conduct than a cohort study, with nearly the same level of precision (p. 90). In our simulations, we found that the cohort design resulted in estimates with moderately greater precision as evidenced by confidence intervals that were, on average, moderately narrower than those arising from NCC designs. Thus, the increased economic efficiency of the NCC design comes at the cost of decreased statistical efficiency.

Essebag and colleagues examined the relative computational efficiency of the NCC design and the cohort design with time-dependent exposures.^19^ Using a single dataset, they compared the computing time required for cohort and NCC analyses with time-dependent exposures. Although the relative increase in computing time for the cohort analysis compared with the NCC analysis was substantial, the absolute differences in computing time were small. In today's era of fast and relatively inexpensive computing power, we speculate that in most settings, the choice between which design to use will not be based on computational demand. We suspect that in most settings that use administrative or other secondary data, the decreased statistical efficiency in the NCC design will result in the cohort analysis being the default approach.

In the current study, we have focused on relatively simple approaches to operationalizing exposure. In two of the three settings, exposure was fixed at baseline, whereas in the other setting, exposure was a binary exposure that occurred once over the course of follow-up. In the applied literature, there is a move to more complicated methods of operationalizing exposure, particularly in settings with time-varying exposures or looking at the recentness of exposure. In such settings, it may be reasonable to use the NCC design for ease of operationalizing exposure, analyzing the data, and interpreting the findings. However, we suspect that such an approach will be accompanied with a reduction in statistical efficiency compared with what would be possible with the conventional cohort design.

There are certain limitations to the current study that suggest directions for further research. First, in the current study, we selected controls from subjects who were in the risk set of the case at the time that the case experienced the event of interest. However, we did not examine the effect of additional matching on other risk factors or confounding variables. Subsequent work is needed to examine the impact of matching on additional sets of covariates. Second, in the current study, we have restricted our attention on NCC designs and have ignored other case-based designs such as the nested case–cohort design.^17^ Subsequent research comparing the relative performance of the nested case–cohort design with that of the NCC design and the cohort design is merited. Langholz reviewed analytical approaches for the case–cohort design and discussed its advantages and disadvantages, including issues of statistical efficiency, in comparison with the NCC design.^29^ Third, we have focused our attention on settings in which the values of confounding variables are fixed at baseline and do not vary over the duration of follow-up. We have not examined estimation of treatment effects in settings in which both treatment and confounding variables vary over time and in which time-varying confounding variables can be influenced by prior treatment or exposure. Marginal structural models have been developed for use in this context.^30^–^33^ Consideration of these types of scenarios was beyond the scope of the current study. We also want to note that, although the most common NCC design is based on the simple random sampling of the controls, there are other sampling designs for the controls such as countermatching design, which is a stratified NCC design.^34^ This design may provide improvement in statistical efficiency depending on the available additional information on cohort members and the type of the study. Other sampling designs for the controls are discussed by Langholz.^35^

In conclusion, we found that, across a wide range of different settings and different scenarios, the use of a cohort design tended to result in estimates with lower bias and greater precision compared with the use of an NCC design.

## CONFLICT OF INTEREST

The authors declare no conflict of interests.

## KEY POINTS

- The cohort design results in estimates with greater precision and lower mean squared error compared to estimates from a nested case-control design.
- In settings with competing risks or time-varying treatments, a simple nested case-control design can result in biased estimation compared to a cohort design.
